# The Need to Include a Fluorine Mass Balance in the Development of Effective Technologies for PFAS Destruction

**DOI:** 10.1021/acs.est.3c10617

**Published:** 2024-02-05

**Authors:** Sanne J. Smith, Mélanie Lauria, Christopher P. Higgins, Kurt D. Pennell, Jens Blotevogel, Hans Peter H. Arp

**Affiliations:** † Department of Water Management, Delft University of Technology, Stevinweg 1, 2628 CN Delft, The Netherlands; ‡ Department of Environmental Science, Stockholm University, Svante Arrhenius Väg 8, 10691 Stockholm, Sweden; § Department of Civil and Environmental Engineering, Colorado School of Mines, 1500 Illinois Street, Golden, Colorado 80401, United States; ∥ School of Engineering, Brown University, 184 Hope Street, Box D, Providence, Rhode Island 02912, United States; ⊥ Commonwealth Scientific and Industrial Research Organisation (CSIRO), Environment, Waite Campus, Urrbrae, SA 5064, Australia; # Norwegian Geotechnical Institute (NGI), P.O. Box 3930, Ullevål Stadion, NO-0806 Oslo, Norway; 7 Department of Chemistry, Norwegian University of Science and Technology (NTNU), NO-7491 Trondheim, Norway

**Keywords:** PFAS, transformation, mineralization, analytical chemistry

Many emerging technologies, including electrochemical oxidation, plasma, hydrothermal alkaline treatment, supercritical water oxidation, photocatalytic degradation, sonolysis, and thermal treatment, are being developed and marketed for the destruction of per- and polyfluoroalkyl substances (PFAS) in environmental media.^1^ In contrast to conventional treatment methods designed to capture and concentrate PFAS, the goal of these technologies is to actually destroy PFAS. As a consequence, there is the potential to generate and unintentionally release transformation products, such as ultra-short-chain PFAS (e.g., C_2_–C_3_) or longer-chain fluorinated compounds.

The importance of transformation (by)product formation during the destructive treatment of PFAS has been recognized in the literature.^2,3^ Nonetheless, studies describing destructive technologies may still report concentrations of only the parent compound or a small set of target PFAS and often confuse “transformation of target species” with “mineralization”. In contrast to that of other organic micropollutants, the degradation of PFAS can be readily monitored by tracing the fluorine element. When technologies are tested in spiked, laboratory-prepared solutions or solids, PFAS concentrations are usually sufficiently high and background fluoride concentrations sufficiently low to allow tracking of defluorination with fluoride measurements. This type of confirmation is often achieved using fluoride-selective electrodes, sometimes verified by ion chromatography (IC) or other methods.

However, to thoroughly monitor potential byproduct formation under field conditions, it is crucial to test these technologies in real matrices. Here, high background fluoride concentrations, low total PFAS concentrations, the presence of perfluoroalkyl acid (PFAA) precursors, and possible precipitation of fluoride with naturally occurring cations may prevent tracking of increased fluoride concentrations as a measure of PFAS defluorination. In this case, it is imperative to include both targeted and nontargeted analytical methods, to understand degradation pathways and close the fluorine mass balance as much as possible. Typically, only academic researchers perform this type of analysis, because commercial technology developers are less motivated to look for nonregulated byproducts. Additionally, the academic sector often has greater access to the necessary high-resolution analytical instrumentation and data processing capabilities necessary for nontargeted analysis.

As a first step, a unification of terminology in the field of PFAS destruction research is needed. As outlined by Horst et al., mineralization of PFAS is taken to mean complete defluorination, regardless of whether the carbon is fully oxidized to CO_2_.^2^ Defluorination of a PFAS requires the release of inorganic fluorine but may still have fluorinated organics (including PFAS) as terminal degradation products, in which case mineralization does not occur. Degradation is simply transformation of a target PFAS into another molecule but does not necessarily include defluorination. To illustrate this distinction, if all perfluorooctanoic acid (PFOA, C_8_) in a matrix is degraded to perfluoroheptanoic acid (PFHpA, C_7_), the technology will have a PFOA degradation efficiency of 100%, a defluorination efficiency of 13% (provided the C–F bonds in the lost CF_2_ moiety are broken), and a mineralization efficiency of 0%.

When applied appropriately, measurement of extractable or adsorbable organofluorine (EOF/AOF) pre- and post-treatment can be an effective way to distinguish degradation from defluorination. In this approach, the organofluorine in a sample is isolated by extraction or adsorption and quantified by combustion ion chromatography (CIC).^4^ CIC is a nonselective technique, but the chosen extraction or adsorption procedure will determine which organofluorine groups are captured in the measurement, which may include non-PFAS organofluorine (e.g., singly fluorinated pharmaceuticals). Common procedures often miss (ultra)short-chain PFAS, which may cause overestimations of the defluorination efficiency. Therefore, a separate extraction and targeted analysis of (ultra)short-chain PFAS may be needed. Other techniques can also be used to measure AOF in adsorbent material, such as particle-induced γ-ray emission (PIGE) or instrumental neutron activation analysis (INAA); however, CIC is best established in fluorine mass balance studies, and its detection limits are 20–40 times lower than those of INAA and PIGE.^5^

Total oxidizable precursor (TOP) analyses can sensitively estimate the concentrations of PFAA precursors that can oxidize to PFAAs, thereby giving more information than only targeted analysis, but may still underestimate the presence of (ultra)short-chain and non-ionizable PFAS.^6^ In contrast, compared to traditional LC-MS-, TOP-, or EOF-based methods, ^19^F NMR is an analytical technology that is well suited for the analysis of ultra-short-chain PFAS.^7^^19^F NMR can also differentiate between organic and inorganic fluorine without extensive sample workup and detect neutral PFASs because it does not rely on ionization. For these reasons, ^19^F NMR is suitable for quantifying the effectiveness of destructive technologies, because fluorinated transformation products cannot be lost during extraction, LC column elution, or ionization. However, NMR typically has detection limits that are ≤5 orders of magnitude higher than those of LC-MS-based methods.

In EOF/AOF, all structural information about the organofluorine is lost, and TOP assays provide limited structural information. To accurately determine reaction byproducts and degradation pathways, high-resolution mass spectrometry (HRMS) is needed, which can be coupled with density functional theory (DFT) simulations and other computational methods. HRMS is a powerful tool for suspect and nontarget screening of PFAS, because it can provide detailed structural information at low concentrations,^8^ particularly in the initial development phase of technologies. Recent advances have improved the annotation of unknown PFAS using nontargeted LC-HRMS workflows; however, direct quantification remains challenging due to the lack of reference standards.

A final challenge in closing the mass balance is the measurement of volatile organofluorine compounds in the gas phase. In thermal technologies, products of incomplete combustion (PICs) can be a significant fluorine sink, and plasma and electrochemical degradation may result in gas-phase organofluorine emissions. Evaluating the potential formation of volatile organofluorine compounds is critical, even when other analytical tools suggest a (nearly) complete mass balance. Some volatile organofluorine compounds are particularly problematic due to their high global warming potential. Many of these compounds can be analyzed by GC-MS, with GC-HRMS specifically being used to identify suspected fluorinated transformation products in the off-gas of destructive technologies.^9^ However, similar to LC-HRMS, reference standards and standardized workflows are needed to achieve more widespread adoption of nontargeted GC-HRMS methods. Additionally, gas-phase sampling is challenging, as many of the gas-phase organofluorine substances may not sorb to commercial filters or resins. Consequently, whole gas sampling becomes necessary, for example, using Summa canisters or Tedlar bags along with established sampling protocols (e.g., US EPA OTM-45 and OTM-50).

**Figure 1:**
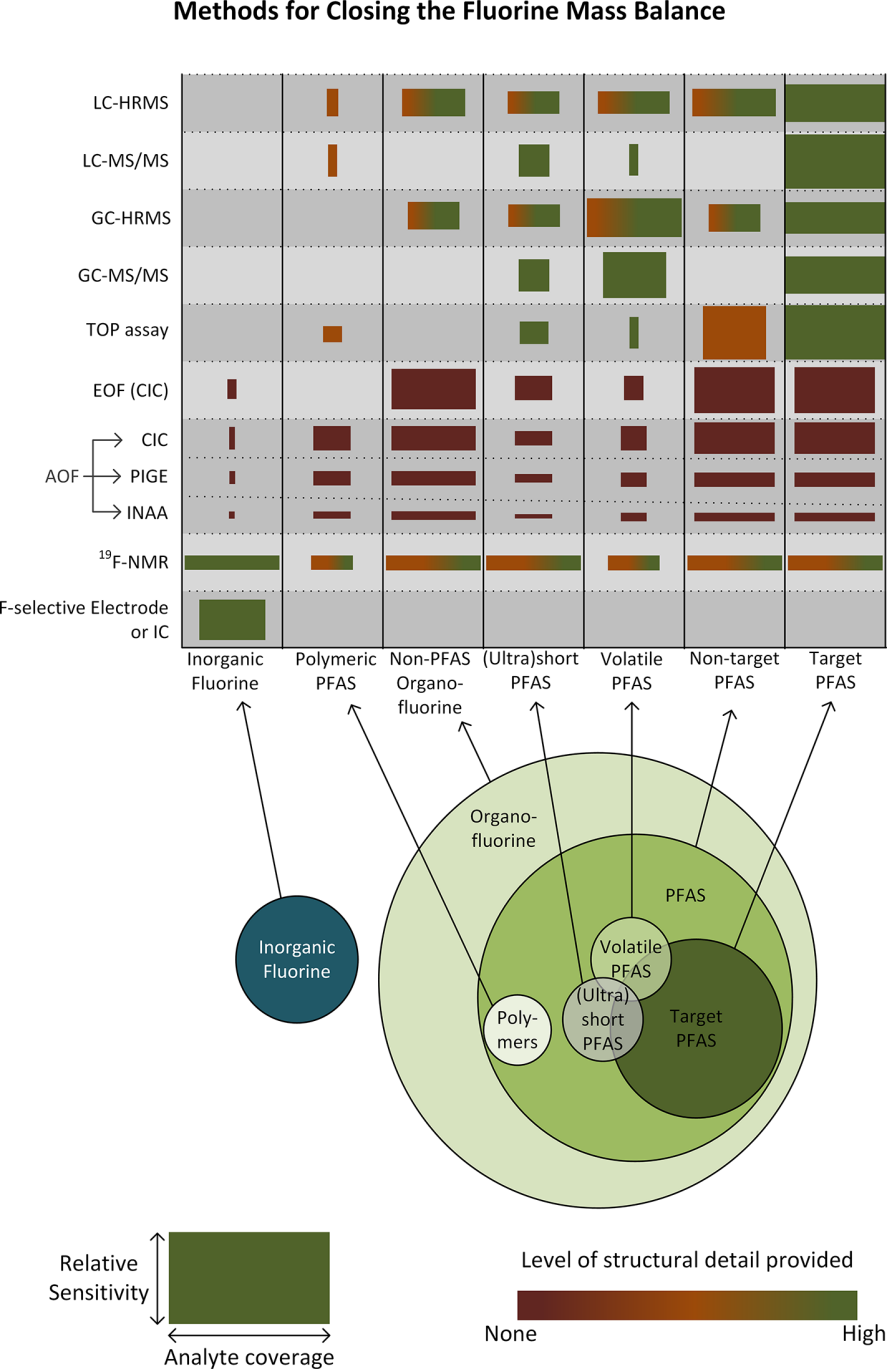
Overview of the relative potential of analytical methods to complete the fluorine mass balance in PFAS destruction, for different types of fluorinated substances potentially found in environmental samples. “Target PFAS” are the typically <100 specific PFAS included in a GC- or LC-MS analyte list, for which standards are available. “Polymeric PFAS” include fluoropolymers, perfluoropolyethers, and side-chain fluorinated polymers and may be present in environmental samples as micro- or nanoparticles. The heights of the boxes represent the general sensitivity of the method as typically employed, and the widths the general coverage of the method within the corresponding group of fluorinated substances. These parameters also strongly depend on the sample types (air, water, soil, etc.), sample collection (grab vs integrated and whole air vs sorbents), and preparation methods (extraction and concentration), which are not included in the figure and can vary significantly depending on the practitioner. Ideally, EOF and AOF methods detect only organic fluorine, but because the extent of fluoride removal is often slightly below 100%, these methods are shown to include some inorganic fluorine.

An overview of the analytical methods that can be used to close the fluorine mass balance is given in Figure 1. Many researchers will not have access to all of these analytical techniques, but we recommend seeking out collaborators or using commercial laboratories with advanced capabilities when possible. Both EOF and TOP analysis are commercially established and should be included in field studies, preferably combined with targeted analysis of transformation products. We also strongly recommend that researchers clearly state the limitations of their studies, instead of “overselling” novel technologies that may result in the formation of harmful degradation (by)products. Arguably, electron transfer reactions in undefined heterogeneous environmental media will always result in byproduct generation, and it is the responsibility of researchers to acknowledge and identify these, to facilitate determination of their risks. Remediation can convert one problem into another, and only through efforts to complete the mass balance and understand the degradation mechanisms can we ensure that destructive technologies are benign instead of regrettable.
